# Virtual Oral Health across Canada: A Critical Comparative Analysis of Clinical Practice Guidances during the COVID-19 Pandemic

**DOI:** 10.3390/ijerph20054671

**Published:** 2023-03-06

**Authors:** Pascaline Kengne Talla, Nora Makansi, Pierre-Luc Michaud, Robert Durand, Paul J. Allison, Elham Emami

**Affiliations:** 1 Faculty of Dental Medicine and Oral Health Science, McGill University, Montreal, QC H3A 1G1, Canada; 2 Department of Dental Clinical Sciences, Faculty of Dentistry, Dalhousie University, Halifax, NS B3H 1W2, Canada; 3 Faculty of Dental Medicine, Université de Montreal, Montreal, QC H3T 1J4, Canada

**Keywords:** teledentistry, clinical practice guidances, dental regulatory authorities, oral care

## Abstract

During the COVID-19 pandemic, teledentistry was suggested as a cost-effective and promising approach to improve access to oral health care. In response, Canadian provincial and territorial dental regulatory authorities (DRAs) published teledentistry-related clinical practice guidances (TCPGs). However, an in-depth comparison between them is needed to understand their gaps and commonalities so as to inform research, practice, and policy. This review aimed to provide a comprehensive analysis of TCPGs published by Canadian DRAs during the pandemic. A critical comparative analysis of these TCPGs published between March 2020 and September 2022 was conducted. Two members of the review team screened the official websites of dental regulatory authorities (DRAs) to identify TCPGs and performed data extraction. Among Canada’s 13 provinces and territories, only four TCPGs were published during the relevant time period. There were some similarities and differences in these TCPGs, and we identified gaps pertaining to communication tools and platforms, and measures to safeguard patients’ privacy and confidentiality. The insights from this critical comparative analysis and the unified workflow on teledentistry can aid DRAs in their development of new or an improvement to existing TCPGs or the development of nationwide TCP guidelines on teledentistry.

## 1. Introduction

Despite the strategies to strengthen healthcare systems and ensure an equitable distribution of resources, oral health inequalities persist and are still a major public health concern worldwide [1,2]. Access to oral healthcare remains a huge challenge for many people [3,4]. The COVID-19 pandemic has further exacerbated those challenges [5] by the restriction of access to oral healthcare, which is affecting patients and the oral healthcare workforce [6]. During the first wave of the pandemic, many governing bodies limited oral healthcare to emergency care, and most elective dental services were postponed to reduce the virus’ transmission [7]. This situation made evident that alternative methods to dispense health care were necessary [8].

Teledentistry is a branch of telehealth that refers to the use of technology to deliver virtual oral health care and education through a variety of modalities including, but not limited to, live video (synchronous), store and forward (asynchronous) techniques, remote patient monitoring, and mobile health by employing several devices such as phones, videoconferencing, and text messaging. [9]. Teledentistry has been suggested as a cost-effective approach to improve access to oral care, regardless of geographical locations [10,11,12]. It brings new opportunities for the practice of dentistry as well as for research and education and the implementation of health policies [13,14,15].

Teledentistry can: (i) improve access to healthcare; (ii) increase communication between patients and oral health care providers as well as among health care providers; (iii) reduce costs for patients and society in general; and (iv) enhance patients’ oral health-related outcomes, experiences, and the quality of care [16,17,18,19,20]. However, despite the advances and expansions in the scope and usage of teledentistry, there are still obstacles at the micro (e.g., sociodemographic factors, mistrust, low motivation, the lack of familiarity and knowledge of technology), meso (e.g., lack of infrastructure and equipment, limited services and time) and macro (e.g., legislative and policy issues, confidentiality and security concerns, reimbursement) levels that can lead to low adoption by oral health care providers and patients [21,22]. Therefore, substantial investments in the governance of digital health, the development of policy, legislations and infrastructures, and the training and development of competencies in digital health are required to support stakeholders in the optimal use of teledentistry [23].

The TCPGs have been published by certain national and regional dental regulatory authorities (DRAs) during the COVID-19 pandemic to shape the delivery of traditional oral care [9,24,25]. These TCPGs are often derived from informal consensus rather than evidence-based data [26] and involve a review and methodological critique of the existing literature without a systematic review of available evidence [27]. Similar to the CP guidelines, the CP guidance could assist practitioners and patients’ decisions about appropriate health care for specific clinical circumstances with multilevel benefits for patients (e.g., improvement of patients’ outcomes and the quality of care), for health care providers (e.g., inform clinical decision making), and for health care systems (e.g., quality improvement activities) [28].

In Canada, the provinces and territories govern health care and services, and each jurisdiction has its own DRA [29]. Some of these provincial and territorial DRAs had published TCPGs during the COVID-19 pandemic. Considering the endemic situation of the COVID-19 pandemic, the growing exposure to teledentistry among dental staff and patients, the relevance of evidence based-dentistry, and the need to develop competencies in teledentistry, it is important to have a better understanding of the scope of teledentistry across Canada. A comprehensive review of existing TCPGs across Canada based on a rigorous approach is needed to optimize current practices and assist with developing future policies. In a previously published environmental scan of the opportunities of teledentistry [30], the authors reviewed the literature on the promotion and the usage of teledentistry since the start of the COVID-19 pandemic until August 2021 in Canada. Although they considered sources such as federal, provincial, and territorial government documents, media articles, and files from insurance companies, the report is descriptive without in-depth comparisons between the documents. The current study addresses this gap and uses a rigorous methodological process to assess the similarities, differences, and gaps between the TCPGs published by Canadian provincial and territorial DRAs. Moreover, our review contributes to the advancement of knowledge on unified TCPGs in order to improve access to oral health care beyond the COVID-19 pandemic. The unified TCPGs could be valuable in developing a Canada-wide approach to teledentistry.

### Purpose

The aim of this review is, therefore, to identify, synthetize, and provide a comparative analysis of the TCPGs across Canada from March 2020 until September 2022, and to evaluate them for completeness, clarity, consistency, and gaps.

## 2. Methods

### 2.1. Study Design

This review involves a critical comparative analysis of the available TCPGs published by Canadian provincial and territorial DRAs. A critical review is a form of narrative literature review aiming to identify, analyze, synthetize, and aggregate the existing literature about a topic [31,32]. This type of review is an opportunity to analyze existing knowledge and provide a conceptualisation of innovation [31]. Beyond the description of existing guidance, the ‘critical comparative’ component of the analysis aims to contrast and summarize the existing recommendations and then propose a unified teledentistry workflow to forge a path for its implementation across Canada.

### 2.2. Study Setting

There are 13 DRAs in Canada, including those in the provinces of Alberta (AB), British Columbia (BC), Manitoba (MB), New Brunswick (NB), Newfoundland and Labrador (NL), Nova Scotia (NS), Prince Edward Island (PEI), Ontario (ON), Quebec (QC), Saskatchewan (SK), and those in the territories of the Northwest Territories (NT), Nunavut (NU), and the Yukon (YT) [29].

### 2.3. Eligibility Criteria

To be eligible, a TCPG would have had to focus on teledentistry and be published (i) by a Canadian DRA; (ii) between March 2020 and September 2022; and (iii) in French and/or English.

### 2.4. Data Screening and Selection

Two strategies were used to collect data from March 2020 to September 2022. First, the official websites of all Canadian DRAs and their newsletters were searched. In addition, the references or links within each document were reviewed to find other relevant publications. These reviewers screened the website of the Canadian Dental Association (CDA) to capture some relevant information. Although the CDA is a non-regulatory authority, it represents Canadian dentists, supports the dental practice, and disseminates information on oral care [33].

The members of the research team who have extensive experience and expertise in teledentistry, clinical practice, policies, and evidence synthesis performed the review procedures. Two members of the research team independently screened the websites to identify all TCPGs and any updates. These two individuals then pilot tested a data extraction protocol with one TCPG. Disagreements in this process were resolved by discussion. A final data extraction spreadsheet was then developed according to the aims of the study.

### 2.5. Data Analysis

A suitable type of analysis for critical reviews is narrative synthesis [31]. Two members of the research team performed the qualitative content analysis. Data were reported in tabular forms for the different components of the published documents (Table 1, Table 2, Table 3 and Table 4). The findings were further analyzed for consistency, similarities, particularities, gaps, and clarity.

## 3. Results

### 3.1. Characteristics Related to Format and Content of TCPGs

Among the 13 DRAs across Canada, only four of them (the Royal College of Dental Surgeons of Ontario (RCDSO), L’Ordre des Dentistes du Québec (ODQ), the College of the Dental Surgeons of Alberta (CDSA), and the Newfoundland and Labrador Dental Board (NLDB) published TCPGs during the relevant period [34,35,36,37,38] (Table 1). The CDSA published its guidance on teledentistry in April 2020 [34] and released an updated guidance in January 2022 [37]. The DRA in Manitoba only referred to teledentistry in an annual report, addressing a “new billing code” for teledentistry and its intent to not adopt said code [39]. The British Columbia College of Oral Health Professionals (BCCOHP), previously the College of the Dental Surgeons of British Columbia (CDSBC) [40], refers to the RCDSO’s teledentistry guidance in the COVID-19 References and Resources section of their website. In the titles of these documents, some specifically use the terms “teledentistry” and “guidance” (ODQ, RCDSO) while others use the terms “remote care” and “guideline” (CDSA). All TCPGs identified were free and publicly available on the websites of each relevant DRA. All the available TCPGs were in English, however, the ODQ and RCDSO had their TCPGs in both English and French.

The primary difference between the guidance was the structure and the format. The CDSA, RCDSO, and the NLDB provided an information sheet with different headings including purpose of use, requirements, and additional requirements. The CDSA further adds information regarding managing patient expectations and billing for remote appointments. The ODQ provided an additional flowchart on the steps for dentists who are treating new and existing patients with or without video communication technologies. The ODQ, RCDSO, and the NLDB provided information on the modes of delivery for teledentistry, while the CDSA did not.

### 3.2. Scope and Purpose

Table 2 presents the scope and characteristics of TCPGs for different DRAs. The TCPGs for each of these provinces were in response to the COVID-19 pandemic and did not authorize the use of teledentistry in any other context or circumstances. 

The use of teledentistry in Ontario, Quebec, Alberta, and Newfoundland and Labrador was restricted to dentists with a license and entitled to practice in their respective provinces to treat patients living in those provinces. However, the CDSA states that dentists can provide care to patients outside their provinces only if they are registered in those jurisdictions. The RCDSO mentions that dentists need not be physically present in Ontario to provide remote oral health care.

The scope of TCGPs was to provide guidance to dentists on the use of teledentistry or its acceptable use to facilitate the remote assessment; the use of remote technology and telephone; to reduce patient and dentist in-person contact; to prevent unnecessary patient visits to emergency departments and community clinics; to assess and triage patient’s oral healthcare needs and to determine the next steps.

### 3.3. What Is Emergency Dental Care?

As the DRAs have specified the use of teledentistry for emergency dental care (Table 2), we explored how each of them defined a dental emergency in the context of the COVID-19 pandemic. Dental emergency situations are mentioned in separate documents [41,42,43,44]. According to the TCPGs, the most common oral emergencies were pain, acute infection, prolonged bleeding, and trauma. The RCDSO classifies it as “treatment needs as those requiring emergency, urgent and non-essential care with dental emergency defined as a potentially life-threatening condition that requires immediate treatment and cannot be managed by over-the-counter medications” [41]. The ODQ refers to a dental emergency as: “intolerable pain; acute infections; significant or prolonged bleeding medically required treatment as a pre-intervention to surgery or cancer treatment; and lesion suspected of malignancy requiring emergency biopsy” [42,43]. Similar to the RCDSO, they too have listed several additional endodontic, prosthodontic, and orthodontic emergencies.

According to the NLDB TCPG, a dental emergency is “a situation which based on the professional judgment of the dentist to determine better if a person needs immediate attention” [38]. The CDSA mentioned that emergency dental treatment is required when the condition cannot be managed by over-the-counter medications. They provided an algorithm for patient triage based on the nature of the emergency and any COVID-19 symptoms and risk factors [44].

All the available TCPGs suggested the assessment of an emergency and provision of pharmacological management, if possible, prior to an in-office physical assessment and treatment.

### 3.4. Definition and Applications of Teledentistry

Table 3 presents the definitions of teledentistry as given by the TCPGs. In their titles, three out of the four TCPGs interchangeably use the terms “teledentistry” and “remote care”. The definition of teledentistry (RCDSO, NLDB, and ODQ) and remote dentistry (CDSA) are common to all TCPGs as the provision of patient dental care at a distance using information and communication technologies. The modalities mentioned are telephone, audioconference, and videoconference. The CDSA additionally mentioned email, texts, other technology/communication apps, and unsecured platforms such as Zoom (Pro or higher), Facetime by Apple, Skype, and Teams by Microsoft for communication. The ODQ indicated that dentists can choose the communication tools they prefer, however, they are strictly prohibited from using social media (Facebook, Snapchat, Instagram, Twitter, etc.). RCDSO and NLDB have not specified the communication tools.

While the NLDB and RCDSO have included “mhealth” (the use of mobile health devices such as smartphones, tablet computers, and smart watches) in addition to consultations on live mode of delivery, store and forward, and remote monitoring patient as the modalities of teledentistry, ODQ and CDSA did not mention it..

All four DRAs specified how to use their TCPGs during the COVID-19 pandemic. In addition, ODQ, CDSA, and NLDB stated that their TCPGs may not be used in any other situations. On the other hand, this information was not mentioned in the RCDSO TCPG. The requirement for the applications of teledentistry varies between provinces. The RCDSO TCPG is to be used to “ensure continuity and ongoing provision of necessary dental care” by prescribing medications, facilitating patient referrals for in-person clinical examination or treatment, and referrals to other dentists, allied health care providers, or to hospitals in case of extreme emergency situations. The ODQ and NLDB TCPGs specified that teledentistry be used for the delivery of emergency dental care, assessing patients’ oral healthcare needs, triaging patients, and planning treatments. The ODQ further states that teledentistry can also be used to support other health professionals in the provision of emergency care. From both versions of the CDSA TCPGs, remote oral health care aims to prevent unnecessary patient visits to emergency departments and community clinics in accordance with their “Guidelines on Emergency and Urgent Treatment” [34].

In summary, teledentistry has been recommended for interim diagnoses, prescribing medication, assessing needs for in-person visits, and for referrals to other dentists or health professionals or hospital emergencies.

### 3.5. Maintaining Privacy

The ODQ, RCDSO, and NLDB provided a series of recommendations to address concerns related to the protection of patient privacy (Table 4). The ODQ requires that dentists use data encryption to protect the privacy of the information shared during virtual oral care. Dentists are also required to explain to the patient the potential privacy issues with the technology and obtain their consent for its use. Moreover, the ODQ referred to the need for the availability and assistance of an authorized person if the patient does not have the necessary knowledge on the use the technology selected. To further ensure privacy, the ODQ also stated that the appointment should be conducted in a private environment so that the patient cannot be seen or overheard by unauthorized persons. The RCDSO and NLDB have also stressed the use of technology with privacy and security settings in accordance with the “Personal Health Information Protection Act” and the “Personal Health Information Act” of their respective provinces along with data encryption.

### 3.6. Participants’ Identity

The four DRAs provided information on how to confirm patients’ identity prior to starting, charting, and keeping record of the virtual appointments, as well as how to maintain privacy. Dentists are required to confirm a patient’s identity with a suitable identity card such as driver’s license/passport. The RCDSO TCPG also mandates that dentists provide their proof of licensure for a new patient (Table 4).

### 3.7. Record Keeping

Table 4 presents the information related to record keeping of a teledentistry session. The ODQ issued elaborate guidance on maintaining teleconsultation records. In addition to the standard details, dentists are required to enter the type of technology used, how the patient’s identity was confirmed, the method used to obtain informed consent, the location of the patient, and any photographs or videos sent by the patient. This information must be entered in the patient’s file after the teledentistry appointment. The RCDSO mandates the record keeping of the teledentistry appointment, mentioning teledentistry as the modality of provision of care and compliance with College’s Dental Recordkeeping Guidelines [45]. Similarly, the NLDB TCPG suggested compliance with the available dental record-keeping guidelines. The CDSA also provided guidance on record keeping, mentioning that if the dentist is unable to access patient records, a signed and dated written or digital record should be obtained, and the details should be saved at the earliest possible moment in the dentists’ charts or software. The original written or digital records must also be saved for reference.

### 3.8. Reimbursement Billing Code

On 31 March 2020, the CDA suggested the use of codes in sub-class 05200 as an interim solution for insurance claims [46]. However, no billing codes are provided by the DRAs, with the exception of the CDSA, which introduced, in April 2020, billing code No. 05201 for remote consultation services using teledentistry. In January 2022, the CDSA updated their TCPG and provided a new code, 08010 [37]. This code could be used for remote consultations of more than 7.5 min (Table 4).

## 4. Discussion

The purpose of this review was to critically evaluate the existing TPCGs published by Canadian DRAs from the beginning of the COVID-19 pandemic until September 2022. The main finding of this study is the limited attention to this new modality of oral care practice, as less than half of the Canadian DRAs have published or referred to any TPCG. In addition, the use of the published TPCGs was restricted to the COVID-19 pandemic. In most of cases, the published TCPGs have not changed over time in comparison to other CP guidances published during the pandemic [47]. In fact, the CDSA is the only province that has published an update and explicitly suggested a billing code for teledentistry. Development of CP guidelines including CP guidance should involve a standard process to reduce variation in clinical recommendations that could affect the healthcare providers’ ability to deliver high-quality care and service [48]. In most cases, there is no mention how the TCPG was developed, the number, the composition, the characteristics of the stakeholders involved, and the frequency of their meetings to prepare the documents. Ideally, groups developing CP guidance must be independence of government, industry, and special interest groups [49]. However, this can be challenging in situations of emergency such as the COVID-19 pandemic where there is considerable pressure and time constraints to produce gold standard guidance [50]. As with most research innovation and other initiatives during the pandemic, they were often developed and performed with a weak level of evidence [50].

Another important observation resulting from this review is the mandate and the position of DRAs, in comparison to the CDA [51]. Similarly, in the United States, there are CP guidelines provided by the American Dental Association [9], but specific guidelines are also provided by each of the states [52]. Interestingly, the state of Ohio requires dentists to obtain a teledentistry permit from the state dental board to provide such services [52]. The CP guidelines published by the ADA were formulated before the pandemic and contain detailed information on the rights and responsibilities of the patients and dentists, the quality of care, the supervision of allied dental personnel, the licensure, and the compensation. They indicate that coverage for teledentistry services should be the same as in-person services regardless of the technology or the location patient and provider [9]. However, reimbursement mechanisms for Canadian physicians vary and are dependent on each province. In Quebec, the same rates and reimbursement codes are used for face-to face visits and virtual care for family physicians [53].

In general, teledentistry was defined in a similar manner by the Canadian DRAs, which published the TCPGs and is in broad agreement with the definitions provided by the American Dental Association [9] and the Australian Dental Association [54]. However, the ODQ and CDSA do not refer to “mhealth”. According to WHO, “mhealth” is a component of digital health and is distinct from telehealth [55]. However, according to the Organisation for Economic Co-operation and Development (OECD), telehealth can include “mhealth” as a means to provide clinical care and monitor patients at a distance [56]. The WHO Global Observatory for eHealth defined “mHealth” as “medical and public health practice supported by mobile devices, such as mobile phones, patient monitoring devices, personal digital assistants, and other wireless devices” [57].

Among the applications of teledentistry that our work identified, the triage of patients and treatment follow-ups were the most common virtual oral health care services. A similar result is reported in the scoping review on the evolution of the teledentistry in Australia [58]. It was observed that the benefits of teledentistry are variable and include its ability to triage patients, provide post treatment follow-ups, and determine treatment plans. Another interesting observation from TCPGs is the relevance of teledentistry for patients who have already been seen by a particular dentist versus patients who are new to a particular dentist. This observation is consistent with the approach used in Australia, where teledentistry is recommended for routine examinations, post operative reviews, and new patient consultations [58]. However, in an overview on teledentistry, Islam et al. [59] reported that it should be used only when there is already a direct relationship between a dentist and patient. Although teledentistry can be relevant for anyone, some people, such as immunocompromised individuals, those living with cognitive and physical disabilities, elderly people in long term facilities, as well as individuals in remote regions without consistent access to dental services, and those for whom the time to travel is a major challenge, could take more advantage of the uptake and integration of teledentistry beyond the pandemic [58,59].

All the available TCPGs stressed patient privacy due to concerns about the possibility of breach of confidentiality and privacy. The RCDSO explicitly mentioned the need to follow the Personal Health Information Protection Act (PHIPA), 2004, privacy and security guidelines for virtual healthcare visits with specific recommendations for health care providers, electronic service providers, and the health information network providers, given by Ontario’s health-specific privacy legislation [34]. The NLDB TCPG refers to the Personal Health Information Act (PHIA), 2012, enacted by the Newfoundland and Labrador legislature [38], while the CDSA refers to the Health Information Act. In relation to this issue of confidentiality and privacy, it is interesting to note the Australian Dental Association guidance’s list on virtual platforms in order of security preference, instructions on managing attendees, setting up unique conference identification and password for each session, and file sharing [54].

There are several challenges relating to the adoption of teledentistry at the political (interest groups), organizational (new roles and workflows), economical (cost and benefits), technical (innovation’ characteristics), relational (interpersonal interactions), and clinical levels as each patient is unique and there needs to be a balance between dentists’ norms and patients’ needs and expectations [60]. For instance, challenges to teledentistry’s implementation can be related to training in teledentistry (for instance, digital literacy, familiarity with the technologies), the absence of guidelines, legal regulation of teledentistry, poor internet connection, and the lack of reimbursement for teledentistry-related activities [19,21,22,25,61]. In addition, teledentistry can have limitations in diagnosing diseases and evaluating a patient’s problems. Special investigations that require a patient’s direct contact and that would aid in an accurate diagnosis are difficult to obtain in a teledentistry session. Patients can have issues with the consistency of interpersonal interactions, the ability to connect to the software, manual dexterity (i.e., patients’ capacity to manipulate screens), and their knowledge of mobile/tablet devices. In the dentist–patient relationship, mutual trust is a key factor for the successful implementation of teledentistry [59]. The lack of clinical contact and interpersonal interaction that is integral to traditional dental care may lead to mistrust and anxiety among patients. Given this, dentists should inform patients of the benefits and challenges related to teledentistry before they schedule a virtual oral care session. For instance, they need to mention potential issues concerning misdiagnosis, treatment, confidentiality, and privacy [19,22,25].

Despite these challenges, teledentistry has many benefits for patients and dental teams, including the capacity to reduce appointment waiting lists, triaging and prioritizing appointments, enhancing communication, and rationalizing the use of time, travel, and costs for all parties [16,17,18,19,20,62,63]. A growing body of evidence reports the potential of teledentistry for remote dental screening, diagnosis, consultation, and treatment planning [9,10]. Several reviews have reported the effectiveness and validity of teledentistry for prevention and health promotion, and its accuracy for diagnosis of oral diseases with results that are comparable to face-to-face interventions [11,12]. Teledentistry has the potential to improve population health [16,17,18,19], patient experiences [13,14,15], and dental health care providers’ (DHCPs) experiences [20]. In addition, there is a high acceptability for teledentistry amongst clinicians and patients [63]. Even with these challenges and benefits, teledentistry is not a substitute for but an addition to in-person oral health care.

An interesting finding is the strategy that is used to increase the awareness of TCPGs. In fact, most of the TCPGs we identified were only available on the websites of the relevant DRAs. There is extensive literature on implementation strategies (e.g., such as passive, active, tailored, discrete, and multifaceted interventions) [64,65,66,67,68,69,70,71] aimed at enhancing the adoption, implementation, and sustainability of innovations in healthcare [68,69,70,71,72] Multifaceted and interactive strategies that involve various stakeholders are most promising in achieving evidence-based practices [73]. For instance, information dissemination through websites could lead to a variable effect, needing alternative techniques to ensure the optimal implementation of guidelines and to overcome the anticipated and current barriers, as well as leverage the facilitators [72].

**Figure 1:**
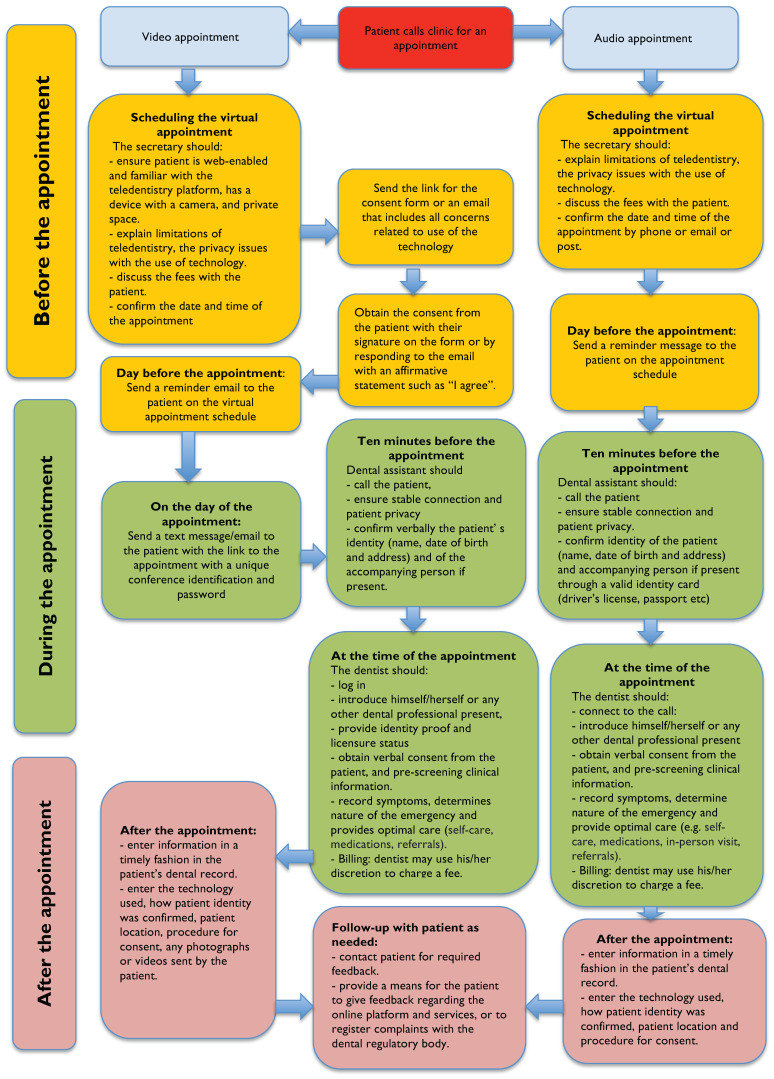
Workflow for the use of teledentistry.

Our results show a gap in Canadian TCPGs due to the lack of specific recommendations for each aspect of virtual oral care. A more comprehensive and clear description of the workflow with definitions of relevant terminologies and best practice recommendations before, during, and after virtual appointments are needed. Clear mention of the roles and responsibilities of the dentist, along with guidelines on the use of different virtual platforms, screening, diagnosis, prescription of medications, billing and coding for teledentistry services and compensation procedures are required. In view of the guidance variations across Canadian DRAs, we have developed a unified TCPG as a reference for any DRA. The unified workflow of a set of tasks suggested (Figure 1) is a combination of material from identified TCPGs, in addition to some information from the relevant scientific literature on teledentistry and telemedicine [51,52,53,54,59]. If consistently performed, the ordered tasks could help streamline the virtual provision of oral health care, enhance the quality of care, and reduce adverse outcomes [74]. This unified workflow is a valuable contribution to the literature and may assist in implementing teledentistry for reasons other than the COVID-19 pandemic, thereby reducing the barriers to accessing oral care.

In addition to implementation strategies and the availability of unified TCPGs, it is important to increase oral health care providers’ confidence in the competencies required for planning, preparing, and providing optimal virtual oral health care [75]. According to the literature [76] and to our knowledge (following consultation in Canadian dental schools), there is currently no specific and mandatory training in information and communication technologies in Canadian dental schools [77]. It has been reported that almost 70% of dentists and dental hygienists attribute a great importance to online courses [78,79]. As the field of digital technologies continues to expand, incorporating teledentistry into the dental curriculum and continuing education programs could therefore provide dentists with the knowledge and skills needed to embrace teledentistry and enhance their attitudes towards it [76,79]. The COVID-19 pandemic has increased the awareness of the advantages of distance dental education [79] as well as the possibilities of teledentistry.

### Strengths and Limitations

The major contribution of this study is the development of a unified TCPG. We constructed this flowchart based on the combined empirical data from the comparison of Canadian TCPGs, in addition to relevant guidelines from countries with similar contexts such as Australia and the United States, as well as Canadian telemedicine guidelines. However, the unified TCPG is primarily focused on the Canadian context. We encourage its adaptation to a specific context as it is not a deterministic tool. This workflow can inform decision makers on the development of TCPG in their jurisdictions or to improve the existing TCPGs. Decision makers, researchers, and clinicians could use the workflow as a guide to inform the development of national TCP guidelines. We believe that this unified TCPG will help researchers, as well as decision makers, to conceptualize the delivery of virtual oral care. It could also be useful in undergraduate and postgraduate education, particularly in promoting the use of teledentistry when necessary.

## 5. Conclusions

This critical review provides valuable insights on the similarities and differences between the TCPGs published by different Canadian DRAs during the COVID-19 pandemic. The results highlighted how these DRAs acted in a timely manner to improve the continuity of care. Despite some differences among the TCPGs, they shared many common features, such as the purposes of teledentistry, communication tools, record keeping, privacy, etc. The results of this review can be relevant to dental education, oral health research, and dental practices and their relevant policies, enabling mobilization of knowledge into practical solutions. The insights gained from our study can potentially aid in the development of a legal framework and inform policymakers on the implementation of teledentistry in their jurisdictions.

This is the first step in the vision for the development of TCP guidelines to improve the accessibility of oral care. According to the literature [49,50], as has been done in other health care fields, the development of optimal guidelines needs working groups involving many stakeholders, such as regulatory and political decision makers, dental representatives, dental hygienists, patients, caregivers, students, and researchers. Future research opportunities will be needed to evaluate the barriers and guide enablers to their adoption and sustainability through oral health care providers. In addition, it is important to identify effective implementation strategies targeting patients, dental health care providers, and oral healthcare systems to promote the optimal adherence to CP guidelines.

## Figures and Tables

**Table 1:** Attributes of teledentistry-related Clinical Practice guidances (TCPGs).

Dental Regulatory Authorities (DRAs)	Title of Document	Province	Target	Fees	Publication Language	Month/Year
ODQ	Emergency Dental Care Using Teledentistry during the COVID-19 Pandemic	Quebec	Patients living in Quebec	Free and available to the public	English and French	April 2020
RCDSO	COVID-19 guidance for the use of teledentistry	Ontario	Patients of Ontario	Free and available to the public	English and French	November 2020
NLDB	Newfoundland and Labrador dental board standards of practice for dentistry in Newfoundland and Labrador	Newfoundland and Labrador	Patients of Newfoundland and Labrador	Free and available to the public	English	May 2020, updated from document published in May 2018
CDSA	Guidelines for Alberta dentists for remote care during the COVID-19 pandemic	Alberta	Patients/residents of Alberta	Free and available to the public	English	April 2020 January 2022

**Table 2:** Scope and characteristics of TCPGs.

Dental Regulatory Authorities (DRAs)	Audience	Indications	Aims and Purpose	Dental Emergency Definition
ODQ	Dentists who are members of the L’Ordre des Dentistes du Québec for the delivery of emergency dental care to patients living in Quebec	Teledentistry in response to COVID-19 pandemic. Not for use in any other context or circumstance.	To provide guidance for Quebec dentists on the use of teledentistry, a practice that has become necessary in response to the COVID-19 pandemic.	The ODQ defines a dental emergency as intolerable pain due to pulpitis, pericoronitis, extensive caries or defective restorations, trauma (fractured tooth with pain, soft tissue lacerations, avulsion, luxation, etc.), acute infections, significant or prolonged bleeding, medically required treatment as a pre-intervention to surgery or cancer treatment needed promptly, lesion suspected of malignancy requiring emergency biopsy. Several endodontic, prosthodontic, and orthodontic emergencies are additionally listed.
RCDSO	Ontario dentists (not required to be physically present in Ontario)	For the duration of the COVID-19 pandemic crisis, dentists should consider the use of teledentistry for the remote assessment, triage, and provision of dental care where possible and appropriate.	To provide guidance to Ontario dentists on the acceptable use of teledentistry for remote assessment, triage, and provision of dental care.	The RCDSO states, in dentistry, an emergency is a potentially life-threatening condition that requires immediate treatment, including oral-facial trauma, cellulitis, or other significant infection, especially if compromising the patient’s airway, prolonged bleeding, or pain that cannot be managed by over-the- counter medications.
NLDB	Dentists who have offices located in Newfoundland and Labrador	Authorised during COVID-19 pandemic. Not authorized for other settings or circumstances not specified.	To provide direction for dentists in Newfoundland and Labrador on the acceptable use of teledentistry during the current state of emergency.	The NLDB defines a dental emergency if, in the professional judgment of the dentist being solicited to provide care, it is determined that a person needs immediate attention to relieve pain, or to control infection, or bleeding that is threatening to life, oral cavity structure, or function.
CDSA	Alberta dentists who are providing remote care during the COVID-19 pandemic	Use of remote dentistry will only be authorized during the COVID-19 pandemic and state of public health emergency in Alberta. Its use will not be authorized in any other setting or circumstances.	To facilitate the use of remote technology and telephone to reduce patient and dentist in-person contact; to prevent unnecessary patient visits to emergency departments and community clinics.	The emergency dental treatment includes treatment of oral-facial trauma, significant infection, or prolonged bleeding, pain, which cannot be managed by over-the-counter medications, or management of known/high risk malignancy.

**Table 3:** Definition and Applications of teledentistry.

Dental Regulatory Authorities (DRAs)	Definition of Teledentistry	Purpose of a Virtual Oral Care Appointment	Modes of Delivery	Communication Tool
ODQ	Teledentistry is the delivery of dental care at a distance, using information and communication technologies. Teledentistry can be delivered through live mode of delivery, store and forward, or remote patient monitoring by a third party.	To prescribe medication, book appointment for in-person visits, referral to other dentists, health professionals, or hospital emergency room.	Live mode of delivery store and forward, and remote patient monitoring (RPM) by a third party	It is up to dentists to choose the communication tool that will be used for sharing information. Communicating with patients using social media of any type (Facebook, Snapchat, Instagram, Twitter, etc.) is strictly prohibited.
RCDSO	Teledentistry is the provision of patient dental care at a distance, using information and communication technologies It involves the use of information and communication technologies to provide care remotely, enables dentists to serve a variety of dental care needs while avoiding close contact with patients. Teledentistry can be delivered through live video (synchronous), store and forward (asynchronous), remote patient monitoring, and mhealth.	To assess and triage existing patients, facilitate patient referral to another dentist for care needs or to allied health care providers for care needs outside the scope of dentistry, or patient referral to hospital for extreme emergency cases.	Live video (synchronous), store and forward (asynchronous), RPM, and mhealth	Audiovisual telecommunications technology, mobile communication devices, such as cell phones, tablet computers, and personal digital assistants (PDA).
NLDB	Teledentistry is the provision of patient dental care at a distance, using information and communication technologies such as live video (synchronous), store and forward (asynchronous), remote patient monitoring, and mhealth.	To assess and triage patient’s oral healthcare needs and to determine the next steps.	Live video (synchronous), Store-and-forward (asynchronous), RPM, and mhealth	Use of technology that will allow dentists to gather information required to proceed with the treatment. If dentists need to prescribe medication to a new patient, technology with audio-video capacity will be required to allow for adequate assessment prior to prescribing medication.
CDSA	Remote dentistry is a patient care provided by using communications technologies such as telephone, email, apps, and videoconference, where the patient and provider are in different locations.	To provide interim diagnosis, prescribing medication, assessing the need of in-person visits to reduce patient and dentist in-person contact; to prevent unnecessary patient visits to emergency departments and community clinics, all of which are part of the Guidelines on Emergency and Urgent Treatment	Not included	Phone, email, texts, and other unsecured platforms such as Zoom (Pro or higher), Facetime by Apple, Skype, Teams by Microsoft, and other technology/communication apps.

**Table 4:** Privacy and Billing issues in a teledentistry session.

Dental Regulatory Authorities (DRAs)	Confirming Patient Identity	Maintaining Privacy	Record Keeping	Billing
ODQ	For existing patient: If using technology without video, patient identity should be confirmed by the sound of his or her voice. If using technology with video, patient consent must be obtained for the teledentistry consultation. For new patient: If using technology without video, and the patient cannot be identified, the dentist should determine the nature of the emergency, obtain the contact of the pharmacist and call him to determine if pharmacological treatment can be undertaken.If using technology with video: Patient’s identity should be confirmed by asking to see a photo ID card (driver’s license, health insurance card, passport).	For patients, the potential issues with privacy related to the use of the technology selected should be explained. Patient’s consent should be obtained so that dentists can ensure that the patient has the required knowledge to use the technology selected or that the assistance of an authorized person is available. The technology that protects the privacy of the information should be collected and transmitted during the provision of care and, if possible, it should be used along with the data encryption; the appointment should be In a private environment (for both the dentist and the patient) where the patient cannot be seen or overheard by unauthorized persons.	Dentists should enter information in the patient’s file as soon as possible after the teledentistry appointment. The entry should include the same information as a standard entry in addition to the technological means used, the way in which the identity of the patient was confirmed, the location of the patient during the teledentistry appointment, and how the patient’s consent to the teledentistry consultation was obtained (verbal or written). Photographs or videos sent by the patient to better illustrate the complaint must also be entered in the patient’s file.	Not included
RCDSO	Dentists have to confirm the identity of the patient and provide the patient with proof of their identity and licensure status (if assessing a new patient).	Using technology that has privacy and security settings in accordance with the Personal Health Information Protection Act, 2004. At minimum, technology must have controls to ensure only the intended patient has access to the appointment and where personal health information is stored and/or transmitted, strong encryption must be used. Conducting the teledentistry appointment in a private environment that will ensure patient information is not overheard or seen by other individuals, and confirming with the patient that they are in a private setting and that the technology they are using is secure.	Appropriate records of the teledentistry appointment should be maintained, in compliance with College’s Dental Recordkeeping Guidelines, and it must be noted specifically that the care was provided through teledentistry.	Not included
NLDB	Dentists should confirm the patient’s identity for new patients.	Dentists are required to use the technology that has privacy and security settings in accordance with the Personal Health Information Act, 2012. At minimum, the technology must have controls to ensure only the intended patient has access to the appointment and where personal health information is stored and/or transmitted, strong data encryption must be used. The teledentistry appointment should be conducted in a private environment that will ensure that patient information is not overheard or seen by other individuals; dentists should confirm with the patient that they are in a private setting and that the technology used is secure.	Appropriate records of the teledentistry appointments should be maintained, in compliance with good dental record keeping guidelines and it must be specifically noted that the care was provided through teledentistry.	Not included
CDSA	Dentists should assess if the information provided to identify or confirm a patient’s identity is enough to proceed with protection of privacy. They should have some verification questions and a scan of the patient’s driver’s license can be used to confirm identity.	Dentists are required to follow-up privacy requirements in accordance with the Health Information Act. Dentists should notify the Office of the Information Privacy Commissioner (OIPC) if they are implementing new administrative practices such as the provision of services via remote care. Nonetheless, dentists will still be responsible for the protection of the patient’s health information.	If the dentist is unable to access patient charts or the records software from the remote location, a written or digital record that is dated and signed should be maintained. This record should be saved in the dentists’ charts or software at their earliest opportunity, with the originals saved for reference.	Dentists may use their discretion to charge a fee and obtain patient’s consent for the fee. Codes in the 08010 series may be used for consultations with patients exceeding 7.5 min, which includes verifying patient identity, informed consent, review of medical and clinical history, assessment of the clinical situation, interim diagnosis, remote management, appropriate documentation, and subsequent follow-up calls.

## Data Availability

The data are available from the corresponding author upon request.

## References

[1] Chari M., Ravaghi V., Sabbah W., Gomaa N., Singhal S., Quiñonez C. (2022). Oral Health Inequality in Canada, the United States and United Kingdom. PLoS ONE.

[2] Peres M.A., Macpherson L.M.D., Weyant R.J., Daly B., Venturelli R., Mathur M.R., Listl S., Celeste R.K., Guarnizo-Herreño C.C., Kearns C. (2019). Oral diseases: A global public health challenge. Lancet.

[3] Watt R.G., Daly B., Allison P., Macpherson L.M.D., Venturelli R., Listl S., Weyant R.J., Mathur M.R., Guarnizo-Herreño C.C., Celeste R.K. (2019). Ending the Neglect of Global Oral Health: Time for Radical Action. Lancet.

[4] Bastos J.L., Celeste R.K., Paradies Y.C. (2018). Racial Inequalities in Oral Health. J. Dent. Res..

[5] Dickson-Swift V., Kangutkar T., Knevel R., Down S. (2022). The Impact of COVID-19 on Individual Oral Health: A Scoping Review. BMC Oral Health.

[6] Stennett M., Tsakos G. (2022). The Impact of the COVID-19 Pandemic on Oral Health Inequalities and Access to Oral Healthcare in England. Br. Dent. J..

[7] WHO Considerations for the Provision of Essential Oral Health Services in the Context of COVID-19. https://www.who.int/publications/i/item/who-2019-nCoV-oral-health-2020.1.

[8] Hung M., Lipsky M.S., Phuatrakoon T.N., Nguyen M., Licari F.W., Unni E.J. (2022). Teledentistry Implementation during the COVID-19 Pandemic: Scoping Review. Interact. J. Med. Res..

[9] American Dental Association (ADA) (2015). ADA Policy on Teledentistry. https://www.ada.org/en/about-the-ada/ada-positions-policies-and-statements/statement-on-teledentistry.

[10] Daniel S.J., Wu L., Kumar S. (2013). Teledentistry: A Systematic Review of Clinical Outcomes, Utilization and Costs. J. Dent. Hyg..

[11] Gurgel-Juarez N., Torres-Pereira C., Haddad A. (2022). Accuracy and Effectiveness of Teledentistry: A Systematic Review of Systematic Reviews. Evid.-Based Dent..

[12] Emami E., Harnagea H., Shrivastava R., Ahmadi M., Giraudeau N. (2022). Patient satisfaction with e-oral health care in rural and remote settings: A systematic review. Syst Rev..

[13] Fernández C.E., Maturana C.A., Coloma S.I., Carrasco-Labra A., Giacaman R.A. (2021). Teledentistry and MHealth for Promotion and Prevention of Oral Health: A Systematic Review and Meta-Analysis. J. Dent. Res..

[14] Roy J., Levy D.R., Senathirajah Y. (2022). Defining Telehealth for Research, Implementation, and Equity. J. Med. Internet Res..

[15] Giraudeau N., Varenne B. (2022). Advocacy for a Digital Oral Health That Leaves No One Behind. JDR Clin. Transl. Res..

[16] Estai M. (2018). A Systematic Review of the Research Evidence for the Benefits of Teledentistry. J. Telemed. Telecare.

[17] Da Costa C.B., Peralta F.D.S., Mello A.L.S.F. (2020). How Has Teledentistry Been Applied in Public Dental Health Services?. Telemed. e-Health.

[18] Flores A. (2020). Teledentistry in the Diagnosis of Oral Lesions: A Systematic Review of the Literature. J. Am. Med. Inform. Assoc..

[19] Kumar P., Banasr A.F., Dragan I.F. (2022). Teledentistry at the Crossroads: Benefits, Barriers, and Beginnings. Compend. Contin. Educ. Dent..

[20] Tiwari T., Diep V., Tranby E., Thakkar-Samtani M., Frantsve-Hawley J. (2022). Dentist Perceptions about the Value of Teledentistry. BMC Oral Health.

[21] Tan S.H.X., Lee C.K.J., Yong C.W., Ding Y.Y. (2021). Scoping Review: Facilitators and Barriers in the Adoption of Teledentistry among Older Adults. Gerodontology.

[22] Estai M., Kruger E., Tennant M. (2016). Perceptions of Australian Dental Practitioners about Using Telemedicine in Dental Practice. Br. Dent. J..

[23] Giraudeau N., Bauer M., Tramini P., Inquimbert C., Toupenay S. (2022). A National Teledentistry Study on the Knowledge, Attitudes, Training and Practices of Private Dentists. Digit. Health.

[24] Werts M., Patel P., Mertz E. (2022). Teledentistry Trends in the United States during the COVID-19 Pandemic.

[25] Tonkaboni A., Amirzade-Iranaq M.H., Ziaei H., Ather A. (2021). Impact of COVID-19 on Dentistry. Adv. Exp. Med. Biol..

[26] Field M.J., Lohr K.N. (1990). Clinical Practice Guidelines: Directions for a New Program.

[27] Woolf S.H. (1999). Clinical Guidelines: Potential Benefits, Limitations, and Harms of Clinical Guidelines. BMJ.

[28] Mentz R.J. (2016). Good Clinical Practice Guidance and Pragmatic Clinical Trials. Circulation.

[29] Directory of Dental Regulatory Authorities & Provincial/Territorial Associations. https://www.cda-adc.ca/en/reg_authorities.asp.

[30] Singhal S., Mohapatra S., Quiñonez C. (2021). Reviewing Teledentistry Usage in Canada during COVID-19 to Determine Possible Future Opportunities. Int. J. Environ. Res. Public Health.

[31] Grant M., Booth A. (2009). A typology of reviews: An analysis of 14 review types and associated methodologies. Health Inf. Libr. J..

[32] Guzys D., Kenny A., Dickson-Swift V. (2015). A Critical Review of Population Health Literacy Assessment. BMC Public Health.

[33] Canadian Dental Association About CDA. https://www.cda-adc.ca/en/about/index.asp.

[34] Royal College of Dental Surgeons of Ontario (2020). COVID-19: Guidance for the Use of Teledentistry. https://www.rcdso.org/en-ca/standards-guidelines-resources/2019-novel-coronavirus/covid-19---emergency-screening-of-dental-patients-using-teledentistry.

[35] (2020). Ordre des Dentistes du Québec Emergency Dental Care Using Teledentistry during the COVID-19 Pandemic. https://www.odq.qc.ca/Portals/5/fichiers_publication/DossierSante/Coronavirus/ODQ_Guide%20Te%CC%81le%CC%81dentisterie%20COVID19_vfinale_1470420_anglais.pdf.

[36] Alberta Dental Association and College Guidelines for Alberta Dentists for Remote Care during the COVID-19 Pandemic. April 8 2020. https://www.cdsab.ca/wp-content/uploads/2020/05/Guidelines-on-Remote-Dentistry_1.1.2020.pdf.

[37] Guidelines for Alberta Dentists for Remote Care during the COVID-19 Pandemic. Effective 1 January 2022. https://www.cdsab.ca/wp-content/uploads/2020/05/Guidelines-on-Remote-Dentistry_1.1.2022.pdf.

[38] (2020). Newfoundland and Labrador Dental Board Standards of Practice for Dentistry in Newfoundland and Labrador. https://nldb.ca/Downloads/Standards-Practice-Dentistry-20201124.pdf.

[39] (2020). Manitoba Dental Association MDA Bulletin Summer 2020. https://www.manitobadentist.ca/bulletins/summer2020/MDABulletinSummer2020.pdf.

[40] BC College of Oral Health Professionals COVID-19 References and Resources. https://portal.oralhealthbc.ca/Pages/covid-19-resources.aspx.

[41] Royal College of Dental Surgeons of Ontario Definitions of Emergency, Urgent and Non-Essential Care. https://az184419.vo.msecnd.net/rcdso/pdf/RCDSO_definitions_dental_care.pdf.

[42] Ordre des Dentistes du Québec Liste des Situations D’urgences Dentaires en Contexte de Pandémie de COVID-19. https://www.odq.qc.ca/Portals/5/fichiers_publication/DossierSante/Coronavirus/Liste%20%C3%A9largie%20des%20traitements%20dentaires%20d%E2%80%99urgence.pdf.

[43] Ordre des Dentistes du Québec Qu’est-ce Qu’une Urgence Dentaire?. http://www.odq.qc.ca/Portals/5/fichiers_publication/DossierSante/Coronavirus/COVID-19%20Urgence%20dentaire%206.0.pdf.

[44] Alberta Dental Association and College COVID 19 Dental Emergency Protocol. https://www.cdsab.ca/wp-content/uploads/2020/03/COVID-19-Dental-Emergency-Protocol.pdf.

[45] Royal College of Dental Surgeons of Ontario Dental Recordkeeping Guidelines. https://az184419.vo.msecnd.net/rcdso/pdf/guidelines/RCDSO_Guidelines_Dental_Recordkeeping.pdf.

[46] Canadian Dental Association (2020). CDA Essentials. http://www.cda-adc.ca/en/services/essentials/2020/issue4/.

[47] Khanna M. (2020). Self-Reported Compliance to Recommended Face-Coverings, during the COVID-19 Pandemic, among Canadian Dentists and Dental Hygienists.

[48] Panteli D., Legido-Quigley H., Reichebner C. (2019). Clinical Practice Guidelines as a Quality Strategy. Improving Healthcare Quality in Europe: Characteristics, Effectiveness and Implementation of Different Strategies [Internet].

[49] Keeley P.W. (2003). Clinical guidelines. Palliat. Med..

[50] Dagens A., Sigfrid L., Cai E., Lipworth S., Cheng V., Harris E., Bannister P., Rigby I., Horby P. (2020). Scope, Quality, and Inclusivity of Clinical Guidelines Produced Early in the Covid-19 Pandemic: Rapid Review. BMJ.

[51] Canadian Dental Association (CDA) Interim Guidance Patient Privacy and the Use of Teledentistry Communication Tools During Covid-19 Pandemic. http://www.cda-adc.ca/_files/about/covid-19/CDA%20Interim%20Guidance%20Teledentistry%202020-05-01.docx.

[52] American Dental Education Association (ADEA) Compilation of State Laws and Regulations Addressing Teledentistry or Telehealth Conducted by Oral Health Practitioners. https://adea.org/uploadedFiles/ADEA/Download/ADEA-Compilation-of-State-Teledentistry-Laws-and-Regulations-StatesO-W.pdf.

[53] (2022). Association Médicale Canadienne, le Collège Royal des Chirurgiens et des Médecins du Canada, le Collège des Médecins de Famille du Canada, Rapport du Groupe de Travail sur les soins Virtuels. Soins Virtuels au Canada: Progrès et Possibilités Février. https://policybase.cma.ca/media/PolicyPDF/PD22-05F.pdf.

[54] Australian Dental Association (2021). Guidelines for Teledentistry. https://www.ada.org.au/Covid-19-Portal/Cards/Dental-Profesionals/Critical-Information-For-Members/ADA-Guidelines-for-Teledentistry-(2).

[55] (2017). Executive Board, 142. mHealth: Use of Appropriate Digital Technologies for Public Health: Report by the Director-General. World Health Organization. https://apps.who.int/iris/handle/10665/274134.

[56] Organisation for Economic Co-Operation and Development (OECD) Health Working Papers No. 129. Empowering the Health Workforce to Make the Most of the Digital Revolution. https://www.oecd.org/publications/empowering-the-health-workforce-to-make-the-most-of-the-digital-revolution-37ff0eaa-en.htm.

[57] (2011). World Health Organization (WHO) Global Observatory for eHealth. mHealth: New Horizons for Health through Mobile Technologies: Second Global Survey on eHealth. World Health Organization. https://apps.who.int/iris/handle/10665/44607.

[58] Poirier B., Jensen E., Sethi S. (2022). The evolution of the teledentistry landscape in Australia: A scoping review. Aust. J. Rural. Health.

[59] Islam M.R.R., Islam R., Ferdous S., Watanabe C., Yamauti M., Alam M.K., Sano H. (2022). Teledentistry as an Effective Tool for the Communication Improvement between Dentists and Patients: An Overview. Healthcare.

[60] Greenhalgh T., Rosen R., Shaw S.E., Byng R., Faulkner S., Finlay T., Grundy E., Husain L., Hughes G., Leone C. (2021). Planning and Evaluating Remote Consultation Services: A New Conceptual Framework Incorporating Complexity and Practical Ethics. Front. Digit. Health.

[61] Irving M., Stewart R., Spallek H., Blinkhorn A. (2018). Using teledentistry in clinical practice as an enabler to improve access to clinical care: A qualitative systematic review. J. Telemed. Telecare.

[62] Wilson G.J., Shah S., Pugh H. (2020). What impact is dentistry having on the environment and how can dentistry lead the way?. Fac. Dent. J..

[63] Aquilanti L., Santarelli A., Mascitti M., Procaccini M., Rappelli G. (2020). Dental Care Access and the Elderly: What Is the Role of Teledentistry? A Systematic Review. Int. J. Environ. Res. Public Health.

[64] Grimshaw J.M. (2004). Effectiveness and efficiency of guideline dissemination and implementation strategies. Health Technol. Assess..

[65] Forsetlund L. (2009). Continuing Education Meetings and Workshops: Effects on Professional Practice and Health Care Outcomes. Cochrane Database Syst. Rev..

[66] Ivers N. (2012). Audit and Feedback: Effects on Professional Practice and Healthcare Outcomes. Cochrane Database Syst. Rev..

[67] Pereira V.C. (2022). Strategies for the Implementation of Clinical Practice Guidelines in Public Health: An Overview of Systematic Reviews. Health Res. Policy Syst..

[68] Giguère A. (2020). Printed Educational Materials: Effects on Professional Practice and Healthcare Outcomes. Cochrane Database Syst. Rev..

[69] Flodgren G. (2019). Local Opinion Leaders: Effects on Professional Practice and Healthcare Outcomes. Cochrane Database Syst. Rev..

[70] O’Brien M.A. (2007). Educational Outreach Visits: Effects on Professional Practice and Health Care Outcomes. Cochrane Database Syst. Rev..

[71] Powell B.J. (2015). A Refined Compilation of Implementation Strategies: Results from the Expert Recommendations for Implementing Change (ERIC) Project. Implement. Sci..

[72] Dobbins M. (2009). A Description of a Knowledge Broker Role Implemented as Part of a Randomized Controlled Trial Evaluating Three Knowledge Translation Strategies. Implement. Sci..

[73] Wensing M., Weijden T., Grol R. (1998). Implementing Guidelines and Innovations in General Practice: Which Interventions Are Effective?. Br. J. Gen. Pract..

[74] Kaufman D.R., Pevzner J., Rodriguez M., Cimino J.J., Ebner S., Fields L., Moreno V., McGuiness C., Weinstock R.S., Shea S. (2009). Understanding Workflow in Telehealth Video Visits: Observations from the IDEATel Project. J. Biomed. Inform..

[75] Rutledge C.M., O’Rourke J., Mason A.M., Chike-Harris K., Behnke L., Melhado L., Downes L., Gustin T. (2021). Telehealth Competencies for Nursing Education and Practice: The Four P’s of Telehealth. Nurse Educ..

[76] Gill S., Soofian S., Lewis S., Vaderhobli R.M. (2022). Incorporating Teledentistry into a Dental School Curriculum. J. Dent. Educ..

[77] Amin M., Lai J.Y., Lindauer P.A., McPherson K., Qari H. (2021). Should Dental Schools Adopt Teledentistry in Their Curricula? Two Viewpoints. J. Dent. Educ..

[78] Paolone G., Mazzitelli C., Formiga S., Kaitsas F., Breschi L., Mazzoni A., Tete G., Polizzi E., Gherlone E., Cantatore G. (2022). One-year impact of COVID-19 pandemic on Italian dental professionals: A cross-sectional survey. Minerva Dent. Oral Sci..

[79] Li B., Cheng L., Wang H. (2022). Challenges and Opportunities for Dental Education from COVID-19. Dent. J..

